# Valedictory Address to the Graduating Class in the Atlanta Medical College

**Published:** 1857-10

**Authors:** Thos. S. Powell

**Affiliations:** Sparta, Geo.


ATLANTA

### Medical and Surgical Journal.

Vol. III.] OCTOBER, 1857. [No. II.

### ORIGINAL COMMUNICATIONS.

### ARTICLE I.

*Valedictory Address to the Graduating Class in the Atlanta Medical College.* By Thos. S. Powell, M. D., of Sparta, Geo.

Gentlemen of the Graduating Class
of the Atlanta Medical College:

To you this is a most interesting and momentous occasion, well calculated to excite in the bosom of each one of you mingled feelings of hope and fear. It is the "commencement" of your Alma Mater, which ends your collegiate career, and opens to you a new life. The dreams of youth are now to be succeeded by the realities of manhood. This hour's ceremony will transform you from the pupil to the teacher. It will transfer you from the class and from association with the taught, to the society and position of the learned. Soon you must bid adieu to each other and to kind preceptors, to enter on the vast and untried ocean of actual life to battle for yourselves. Here you are now about to embark on that mysterious ocean, which he alone can navigate successfully who has the requisite skill to avoid rocks upon the one side and whirlpools on the other. No wonder, then, that on such an occasion emotions opposite and conflicting mingle in the bosom of those personally interested. Far be it from me, however, in the present address, to repress your buoyant hopes, or darken your bright anticipations of success; but I feel that I would not faithfully discharge the duty which the kindly confidence of your preceptors has required at my hands, the duty of offering the last counsel which, as a class, you will receive, if I did not tell you that this calm sea which lies so invitingly before you, and on which you are soon to launch your bark, has concealed beneath its waves dangerous rocks; and I tell you, too, to shun them, will depend upon the cool and wise caution with which you will steer your course. Error in judgment with the helmsman of a vessel, or in the conduct of the captain, will prove ruinous to its interest and often disastrous to its crew. So it is with the physician, who acts the part of both; hence, you see the importance of being properly qualified to discharge all of your duties, and surmount the difficulties that await you, if you desire to steer safely over this impetuous stream upon which you are to launch to-day, and ultimately sit upon the summit of usefulness and renown, and descend to the tomb "full of years and full of honor."

It is not my intention to lecture you on the present occasion upon the history of medicine, the advantages or disadvantages of the various theories that have sprung from the broad plain of philosophical research, or from the wide fields of medical speculation. That office has been spared me by those to whom you have listened for the last four months; and in whatever they may have failed to instruct you, for want of time, I refer you to the inexhaustible sources of knowledge to be found in the standard books of the profession. They furnish sources for acquisition in all its departments of learning far more accurate and lucid than any dissertation comprised in the condensed form of an address. Nor is it my object, young gentlemen, in standing before you and this brilliant assemblage of talent and worth, to exhibit boquets gathered from the garden of literature. Far from it; my object is not to gain laurels for myself, but to do you good. And I trust, as my effort must be prepared amid the avocations of a professional life, affording few hours of leisure for such pursuits, will be appreciated and command your approval, or at least a mitigation of your critical severity; for I feel that I speak the truth when I say that no one feels a deeper interest in the prosperity and elevation of the profession than I do—a prosperity in which I trust you may participate—and an elevation which I trust each of you may be instrumental in aiding to achieve.

The topic to which your attention is solicited for a few moments, is the moral and professional duties of the Doctor, with a few suggestive remarks upon one of the great obstacles to his usefulness and success. The science of medicine is peculiar and diversified in its character, differing materially from all others. In its theory and practice it is vast and varied, embracing all the elements of art and science. Unlike Law and Theology, which seek but one element of man's happiness, it includes the province of these and every other science, so that in its principles and practice it comprehends in one vast field the physical, moral and social welfare of the human race. Man's whole moral and material organism—soul, body and mind—are embraced in this varied, beautiful and comprehensive science. The subject, therefore, is of great interest to you, and a matter of vital importance to society at large; to every individual in the country, from the man in the most exalted position in life, to the most humble artisan in the land. Is it not so? most assuredly it is. The public could not do without you if it wished. Then, if this be so, gentlemen, oh how much of the happiness and safety of your fellow-creature depends on your perfectly understanding your varied, arduous and responsible duties.

In order that I may more clearly present the subject to your mind, I propose very briefly to speak of it under two heads—

1st. *The moral duties of the Doctor:* By moral duties, I mean the duties which, on account of his most frequent intercourse with all conditions of society, are frequently required at his hands—duties which are required of him as a citizen and a man, because his duty as a physician has placed him first in the breach where these duties are most required; yet, all men, emmbers of society, are under like obligations.

2d. *The Professional Duties:* When I speak of the Doctor, I mean an educated, learned man, as the word originally meant; one whose intellectual faculties have been developed, disciplined and enlarged. The duties of such a man to society, I desire profitably to depict to you. That mind is power, that superior intellect is inherently powerful, and influential for good or evil, is a truism, and needs no argument to prove. That the doctor is a man, and that man is not only an individual, but a member of society, will also not be denied. That he was not made a "living soul," to idle and dream away his life, however harmless and inoffensive it may be, but to devote the energies of his mind and the virtues of his heart to the discovery and extension of human happiness and knowledge, is authenticated by Holy Writ, and cannot be denied. That individuals depend upon society for protection and security, and society depends upon its component parts for its own stability and happiness, is also acknowledged by all in this age of moral and scientific improvement; therefore, every man has duties to society, to his fellow-creatures, and to his country to perform, and the importance and magnitude of these duties, and the consequent binding obligation to discharge them, greatly depends upon his intellectual progress, and the general condition of the country in which he resides. This will not be contravened, we presume, by any one; nor will it be denied that the position which the doctor occupies in society generally, is one of commanding influence. They are generally educated and intelligent; their pursuits are such as bring them into intercourse with all classes. Speaking as one of the public: we are compelled to take you into our confidence, and often open to you the most secret recesses of our hearts; we tell you what we would disclose to no one else on earth; we pour into your ear accents of anguish almost unutterable. You are, in truth, the true benefactors of the land. Yes, you belong to the true aristocracy, if such a term is applicable to any who live under a republican government. We do not use it in an offensive or braggadocia sense, but we repeat, you are the true aristocracy, that of intellect. You can, to a very great extent, control the morals of the country. Your vocation brings you to a knowledge of the great sources of vice and crime, and all the thousand and one evils that afflict society, and stand in the way of social progress, of individuals and national prosperity. Thus, you see, you are armed with power really formidable for good or evil. You may serve and bless, exalt and depress, reclaim or corrupt. You may often allay and check deeds of violence; sooth down the spirit of party, and strengthen the bands ot social intercourse. Are these outlines of your influence overdrawn? is there any exaggeration tending to inflate you with a false sense of the importance of your position and duties, or to discourage you by a disheartening fear that you are not and cannot become sufficient for the task? Here, young gentlemen, are sitting before you, your professors, the heads of the profession; there are also present some of the most distinguished of the legal profession, who belong to the same aristocracy, and whose duties, responsibilities and field for usefulness, are very much the same. I ask them, am I overstating the case? I imagine I hear them say No. Well, then, if this be so, oh what a responsible, noble, heaven-born profession you have entered, demanding the constant exercise of all your energies in the relief of misery, suppression of vice, and the promotion of virtue. You will thus perceive that you have a great influence, and that no one can deprive you of it; that as educated men you will have with you at all times a tremendous engine of power; hence, you will also see the importance for the good of society and your own happiness, both in time and eternity, that it should be exerted in that way that will accomplish the greatest amount ot good. Remember, gentlemen, the great amount of good which you can be the means of affecting in your daily walk and practice of your profession. Your education, your position, your profession arms you with power for good or evil, which is wonderful and even fearful to contemplate. It is a principle in the moral government of the world, that a good or an evil deed may have no end in its influence on time and eternity; and that also this influence, according to its nature

"for good or evil," goes on progressively increasing and widening throughout eternal duration. Hence, a deed of charity, however small, a kind word or gentle reproof spoken in season, may result in good, and that good begets other good, which acts on some other mind, and that mind may act for good on a thousand other minds; so, the good influence is going on multiplying, widening and increasing throughout all this great and mysterious net-work of creation; and thus, a good word, or one good act, trifling in appearance, may go on through time, extending in its influence, until every human being under the sun shall feel its effects; until every nation and tongue and kindred in time and eternity shall rejoice in its multiplied blessings. Eternity will reveal to our astonished vision what the world has suffered by our neglect of one good act, or the commission of one bad deed. It will unfold, too, before the gaze of an assembled universe the tremendous, and glorious, and eternal good which has resulted from the smallest and most insignificant action in life.

Hence, gentlemen, in this solemn and awful view of the subject, you can appreciate more fully the fearful responsibility that rests upon you, and the importance of wielding all your mighty power for good and glorious ends. Then go forth determined not to neglect the smallest act of kindness or of duty. Go forth on the great field of your labors, with a heart to feel and a soul to sympathize, and a hand to give among all the poor and afflicted of the human race. Do this, and you will achieve your own fame in the undying blessings of your fellow men. You will show forth the glory of God, and carry with you to the great day of final reckoning a reward whose glory and bliss eternity alone will reveal.

If I have succeeded in impressing your minds with the truth that the educated man is powerful in his influence, particularly when his vocation, like yours, brings him in constant contact with many who are less favored, my first object has been accomplished.

I will now proceed to speak briefly of what I conceive to be some of the moral duties of the doctor. I have said that the physician had to mix and mingle with all classes and grades of society; therefore, it is a moral duty you owe to yourselves and to society, that you continue to study, to cultivate and fill your minds with useful knowledge. You should recollect that your education is not completed, but just begun, simply a good foundation, on which you alone, by incessant study and toil, must erect the superstructure. There is no bounds to knowledge; hence, the necessity of pursuing it industriously by study. The more knowledge you acquire the more you will desire, and the larger will be the sphere of your influence and usefulness.

This is an age of science and progress in everything that can enlighten the conscience, and shed light upon the understanding, and give vigor and power to the mind; therefore, if you would become what you should be, and what you will be expected, (a leader in society,) you must study, study everything, all the known sciences, physical and moral; also, the science of human nature.

This is your moral duty, because it is one of your first duties to prepare for exercising the greatest amount of influence for the happiness, advancement and prosperity of society.

This great end can only be accomplished by the united powers which legitimately belong to a highly cultivated mind and morals. Away, then, with the false idea that the physician cannot and ought not to be a man of letters. It is slander circulated by envious, sneering charlatans, who have entered the profession for the emoluments alone, because they were incapable of making a livelihood in any other respectable way, who are willing to creep into and all through their profession like a mere beetle, content to be mere "hewers of wood and drawers of water;" skilful enough when they learn to apply a few general remedies, according to special directions, without being able to command scarcely any resource of a literary character. If, then, young gentlemen, you desire to be what God intended you should be, "the highest style of man"—if you wish to follow the example of your divine exemplar, who went about doing good, healing the sick, relieving the distressed, and comforting the poor. You must not be content with being well versed in those sciences strictly medical, or those that have a direct bearing upon medicine ; but you should as far as your time will permit, acquaint yourselves, with the general principles of every branch of science and literature.

The mind, like the body, not only requires strength for usefulness, but decoration for effect. The human mind may be naturally great and gifted ; it may be richly stored with all the principles of science ; in all its natural powers it may in the study of great truths, grow up to a giant strength ; yet, if it is void of those pleasing ornaments which charm and attract the world, it is less powerful for the performance of good deeds and the achievement of great ends. The mighty Sampson, when shorn of his flowing locks, could not resist an infant's arms. So the most giant intellect, destitute of its mental charms and moral beauties, is quite powerless for victory before the Philistines of ignorance and the host of darkness. To be useful and effective for good, the mind must be not only powerful in strength fully developed, but clothed in those polished charms of decorated beauty which please and captivate the world. Indeed, we see this principle of blending the useful with the ornamental, running harmoniously through the whole economy of nature. There is nothing in the natural universe which does not teach this principle. The great globe in its massive strength, is decorated with living green and flowers of every hue. The mighty ocean, whose heaving bosom bears up the navies of the world, presents its mountain billows, its crested waves, its silvery surface to the enchanted eye. The storm-cloud, before whose fury the mighty forest bows, roars in its thunders wild, and revels in its lightning flash. The great heavens has spread her blue canopy over us, bespangled with ten thousand stars, shining with the light of distant worlds. Thus does all nature, in performing her great designs for the happiness of man, blend in one beautiful whole the useful and the ornamental, the beautiful and sublime. Thus, you see, it is your moral duty never to imagine that you have exhausted the fountain of knowledge ; that you can recline yourselves on beds of indolence and ease, but you must trim the midnight lamp, and continue to drink deeper and deeper of the Pierian spring. To press on, until you have adorned your name and character with that true wisdom and knowledge which shall make you at once an ornament to your profession and a blessing to mankind. A life of study lies before you, and you must enter on it with cheerfulness and decision if you would aspire to real greatness. Distinguished men made so by application, are far more numerous than distinguished men made so by genius.

The influence which you exert on public sentiment is not only by the power of your superior intellect, but by the opinions you utter and examples you set; therefore, it is your moral duty not to indulge in giving utterance to sentiments, which would encourage vice and immorality; but, on the other hand, your conversation in your daily intercourse with your fellow man should be characterized by sentiments of pure morality, and your example should be of the most elevating character, such as would evince great proof beyond the shadow of a doubt, that your judgment and your affections are all enlisted in behalf of right and virtue. You should identify yourselves with the refined, the intellectual and the benevolent, and thus take the proud position to which your education and your profession entitle you. To such a doctor too many thanks can never be given. The value of such a doctor for the public weal can never be estimated by any finite mind. And there might probably be more of such physicians, but for the false and degrading doctrine that the doctor must take no part, in any public or private scheme; or, in other words, he must do nothing but visit his patients punctually, dose them well, charge them less, and live only for selfish ends. How absurd. This is true of no one, much less is it true of the learned, the educated and the refined doctor, who has so many opportunities of doing good and in so many ways. Then let me beg of you never to allow such unphilosophical and unscriptural views to interrupt you in the discharge of your moral duties. Not only as one of the leaders of society, but as an intelligent citizen, you may rest assured that in every good enterprise God will be on your side, and success will be yours. The moral duties of the doctor, are too numerous for all to be presented in a short address. Indeed, it would be necessary in the most elaborate discussion, to select a few of the most important, and even then much would remain unsaid ; but if it was all said, and well analyzed and sifted, it would amount to about this—continue to cultivate your intellect and the moral regulations of your heart, by studying everything that would strengthen, enlighten, and adorn the mind, elevate the standard of morality and virtue; always be ready to promote and encourage virtue and suppress vice; always be willing to aid in carrying out glorious plans for the public good, based on the mighty and immutable rock of christianity.

This, young gentlemen, brings me to the second head of my subject—your professional duties. Some of you may be ready to exclaim, as it has been by Dr. Samuel Johnson, that they are limited, consisting merely in a melancholy attendance on misery ; "a mere submission to peevishness and continual interruption of pleasure." If so, you enter your profession with inadequate or unworthy views. You have only to consult your hearts to be convinced of this. The proof is to be found in the main object of your profession, which is to add to man's happiness by healing him in sickness, and comforting him in affliction. To carry out these objects, you will perceive it involves much. A vast field is open before you, so wide and extended, it will be impossible for me, without occupying more time than prudence would dictate, to do more than take a rapid and cursory view of a few of the most important divisions of the subject, as laid down in the code of medical ethics, adopted by the American Medical Association.

First, your duties to the public—your patients. It is your duty to make yourselves thoroughly acquainted with every branch of your profession. I am willing to admit that many, by simply learning to answer a few general questions in Anatomy and Physiology, and how to apply a few general remedies, may do some good and acquire quite a reputation, particularly if they are adroit in intrigue, as it is said of some of the political demagogues, and can draw to their aid, for the purpose of getting practice, the influence of personal friends, the wealth of relatives, or prejudices founded on political sympathies. But to be a skilful and safe general practitioner, you must have a good knowledge of the structure and functions of every organ of the body. You must know them in health and disease. You must know the causes which derange and disease these organs; and, to do this, you must make yourselves thoroughly acquainted with Anatomy, Physiology, and, in fact, every branch of your profession. This is a duty you owe to the public and your patients. At times of great moment when, there will be no time nor opportunity for inquiring into your competency, you will be sent for, and must act with no other evidence to society, that they can employ you with safety, but the "indicia" of your calling which hangs and glitters over your door. Thus you perceive, young gentlemen, your first professional duty is to study your profession, understand it, keep pace with its improvements, be at all times ready to give to the afflicted all the benefits and advantages that it affords. It is also your duty to be ever ready to obey punctually the calls of the sick. You should be humane, benevolent, gentle and kind to all, but especially to the poor, who have no worldly means of remunerating you for your professional services. Shun the base example of some physicians who, in the hour of deep distress, turn their backs on the afflicted poor, to visit and court the influence of the rich nabob, with his cattle upon a thousand hills. Shun the example of him who attempts to drag down your noble profession from its high moral province to make it one of mere dollars and cents; "who heeds not the cry of the widow and the wail of the orphan," because the glisten of gold does not allure him to their lowly hut. Remember, that to comfort and heal the afflicted part of our fallen race is your highest and noblest duty; remember, too, that when you have thus healed and comforted the poor, your reward is rich on earth, yet far richer in heaven. The pearly tear of gratitude that steals its way down the poor afflicted beggar's cheek, is worth more to you than the brightest gems which sparkle in the starry crown of king or emperor. They will be treasures laid up for yourselves in that brighter and better world, where sickness and sorrow are felt and feared no more. Remember, then, young brethren, that your services (if rendered with the proper spirit) to that class of the human family that the Saviour of the world said we would have with us always, will not be lost, but will be to you the brightest recollections in the history of your life, especially in the hour of death.

You will have to bear much from the ignorance and frailties of others, but for your own and their good, you should keep a constant watch over your tempers, and exercise patience and charity towards them, remembering that the human body is not a piece of senseless machinery, and how seriously their interest may be effected by what you say or do. It is therefore, your duty to avoid all things which have a tendency to discourage your patients or depress their spirits. For your good and the dignity of the profession, it is your duty to guard against everything like coarseness and vulgarity, and undue forwardness, which is, in this day of progress, always offensive, particularly, to the refined, the sensitive and the fastidious, and which will disencline them to employ you, though they may think you highly competent in point of knowledge and skill. It is your duty never to give publicity to anything which is only known to you by the peculiar necessities and privileges of your profession. You are ex-officio the bosom confident of every person who becomes your patient; therefore, you should hold inviolate whatever comes under your notice, in your professional intercourse. Especially avoid the habit of referring to your cases in mixed crowds; never detail the occurrence of the sick room, however trivial or unimportant they may seem. There are some who, in order to gain the reputation of having a large practice generally, and perhaps the practice of some particular families, are in the habit of detailing the symptoms of every case they have with great minuteness to those who can feel no interest in such things, except of idle curiosity. I've no idea there is one before me who will so far forget his own self-respect, or the dignity of his profession, as to follow such examples, yet it is well to warn you lest you may at any time even seem to commit such fatal and disgusting errors, and thereby wound and betray the most sacred feelings of your patient, and degrade yourself and your noble profession, in the opinion of all educated and refined people.

Dr. Rush said disease was a lawless evil. This is true, and to understand its nature, and to meet promptly every change which may take place, it is your duty to visit your patients frequently, watch their symptoms, and accommodate your remedies to them and not to your convenience. Often the cure turns upon a remedy being used not only on a certain day, but a certain hour. This is particularly true in reference to typhoid fever. You will often find it necessary to cure such patients, to visit them at all hours of the night; therefore, it is your duty to do so, however great may be the sacrifice of selfish ease and convenience. This is especially important to young physicians; they have the leisure to make these visits. The oftener they see their patients, the more they know and learn of the character and nature of the disease, the effects of medicine administered; and, aside from this reason, it is your duty to visit them frequently from a proper sense of your awful responsibility. It will not afterwards avail you as a defence, when you are charged by a disappointed husband or wife, that you had relied on his or her statement, if you had the opportunity of ascertaining the correctness of them, but neglected to do so merely because it would have cost a sleepless night or a long ride. I caution you then, young gentlemen, as a general rule, never to rest satisfied with, nor act upon, the mere representations of the friends of your patients, when you can possibly ascertain how the facts themselves really stand by a personal examination. And above all, eschew even a tendency to superficial examination, look patiently and thoroughly into the symptoms and pathology of every case you are called to treat, or upon which you may be consulted, ever remembering that though there is no earthly tribunal before which your conduct and treatment may be called in question, that the great Judge of the universe is at all times rigorously scanning your acts and motives, and will in the great day of final reckoning, hold you responsible for the non-performance of any and all of your duties to the afflicted which have been entrusted to your care.

We could occupy much more of the pre.ent time in enumerating your duties under this head, but we forbear, as there is another branch of your duties, the proper knowledge and due discharge of which is a matter not only of great importance for your own happiness, but often for the good and safety of the sick. I allude to your duties to your professional brethren. As I said in the outset, to-day you enter the profession in full membership. Your diploma will entitle you to all of its privileges, and also obligate you to use your best efforts to support its dignity and preserve its honor, to elevate its standing, and to widen and extend the bounds of its usefulness. Therefore, it is your duty to stand by one another, never allow yourselves to utter a disparaging remark concerning your brethren, either as a body or individually, and especially before those who are or have been patients of such brethren.

If you should be called to consult with a brother, it is your duty to act, towards him, just as you would have him act towards you, were you and he to exchange places. If you should discover any new feature in the case, or error in the treatment, you should avoid exposing it to the profession, or the public, unless compelled to do so by some foolish obstinacy, on the part of the attending physician. Remember that you may one day find yourself placed in the same dilemma, and would wish to be in the hands of a considerate and honorable gentleman ; then never speak, in society, a word against any of your brethren, except in such notorious cases as make it necessary, to maintain the dignity and honor of the profession. Always, as far as you can, conscientiously, act with a charitable forbearance, and in the full spirit of the following beautiful lines:

> "Teach me to feel another's woe
> To hide the fault I see,
> That mercy I to others show,
> That mercy show to me."

Again, it is your duty to be polite to your brethren, especially, when they are on a visit to your place of residence. Then you should call and see them, show them that you recognize them as brethren, and as far as your time and circumstances will admit, endeavor to make their sojourn in your city or community agreeable and pleasant. This is a duty you owe to yourselves and to the profession, and I will venture to give it as my opinion, that the failure on the part of eight tenths of the profession, to discharge this duty, is one of the great reasons, why the profession, has no more of the public confidence and the love and support of its members as a State or National Association. For example, after you, young gentlemen, have practiced a few years, acquired a reputation for science and skill, you visit Mobile, Charleston, Baltimore or Memphis, spend several days in each place, meet the physicians of the city, are introduced, you receive no attention from them, scarely common politeness, I ask you, to put the question to yourselves, whether you would have more or less love for the profession, whether you would sacrifice more or less to maintain its honor and dignity, whether you would have a greater or less desire to attend State Medical Societies? Your answer is obvious, and hence, I repeat, it is a duty you owe to yourselves, as educated gentlemen, a duty you owe to the profession, thus to be polite, kind and accommodating to your brethren. In union, cemented with love, there is strength. But there cannot be, nor should not be any union, only on the grounds of equality.

Such, gentlemen, is the best I am able to present, of the nature of your moral and professional duties. How feebly and imperfectly the exhibition has been, none know better than myself.

My object, as you perceive, has been to deal in plain truths, and not to make a display of learning. I have endeavored to tell you a few of your high duties, and to impress, upon your minds, the necessity and manifold advantages of education and science, in facilitating your discharge of those duties. But I must not pursue this topic further. Already, more time than I intended, has been occupied, but the subject is one of vital importance, and replete with suggestive thought. Take it, young gentlemen, study it, make yourselves masters of it, and resolve, to-day, to live up to its highest and holiest principles.

Lastly. It is to be observed, that your path to success and renown in your profession is, in fact, strewn with a thousand difficulties. They are so various in character, and so great in number, that it requires nothing less than a lifetime of professional service, to unfold them to you, in their proper order and magnitude. Allow me to mention, however, one of the greatest difficulties with which you will have to encounter, and a few of the ways in which it presents itself, namely: The ignorance of the public in regard to their duties to you, and the nature of medicine. This ignorance is manifested often in the manner in which they treat their family physician and in the great quantity of nostrums they buy, and the peculiar pleasure it affords them to give credit to the smallest possible good effects produced by them. For instance, if a member of a family should be slightly benefitted, by taking a few bottles of some celebrated bitters, the one thus benefitted and the whole family, would take special pains and the fearful responsibility to recommend it in every form or stage of any and all diseases, when they would, from their irresistible scruples of conscience, never recommend their family physician, whom they know to be a man of science and skill.

This ignorance is seen again, in a preference which is not unfrequently shown by intelligent citizens, for the uneducated, vulgar, rough, unrefined, drunken physician, to the educated, sober, polite and accomplished practitioner. This assertion will doubtless surprise many, but nevertheless it is true, and I

will venture to assert that there are few communities, even in this age of intellectual improvement, that do not furnish proofs of its truthfulness. I once knew a very illiterate quack to have the entire confidence of quite an intelligent community. In a conversation with one of his neighbors, on the merits of physicians, he said he believed his neighbor, Dr. ——, was one of the best, safest physicians of his acquaintance, that he had his entire confidence. I asked him what his confidence was based upon. Well, said he, he is a self-made doctor—all that he knows is from experience, and you know that is said to be the best teacher, and his cures, sir, are so remarkable; about two months ago, he cured a young lady a few miles below me, after he said she had thrown up six coats of her stomach. I admitted that that was one of the most remarkable cures that had ever occurred in the history of medicine—that Christ himself, while he sojourned upon earth, wrought no cure or miracle superior to that. For a person to live after throwing up the whole stomach, and three coats more than the most scientific microscopic anatomist has been able to discover, was more than God could do under the present arrangement.

This ignorance also shows itself in the way the people suffer their minds to be influenced in forming their opinion of a physician's skill. Some judge of his qualifications by his dress; some by his manners—either polite or eccentric. I have in my mind, at this time, a physician who enjoys the confidence of a large portion of the intelligent citizens of one of our Southern cities, who owes it almost entirely, so say those who know him best, to his exquisite politeness.

It is said that "Dr. Radcliffe, of London, Dr. Pitcairn, of Edinburgh, and Dr. Butler, of Cambridge, owed much of their reputation and business to their eccentricity of manner and conduct." Some judge of his capacity by the magnified and disgusting importance which he may give to the most simple diseases, and his minuteness and great caution in inquiring into the various symptoms, particularly when called to consult with the family physician. Some judge the physician by being able to observe the character of disease at a mere glance at the countenance, affecting an intuitive knowledge that is rarely if ever possessed. Some allow their minds to be influenced by his religious or political creed; some by the position and influence of his family and of those who patronize him. This is discouraging enough to the truly scientific physician; but this is not all. The ignorance of mankind of the nature of medicine is seen in another way, which is still more perplexing than any we have mentioned. They not only confide their health and lives to quacks and unqualified physicians in preference to scientific and truly skilful ones; but we often see very good and otherwise sensible people, who place implicit reliance upon what they call spells and charms to cure their diseases. For instance, to stick your finger through as many holly leaves as you have warts, has long been a sovereign remedy with many people in Georgia. One who has killed a mole in his hand, it is believed by many, has the power to discuss any "rising" simply by passing his hands over it a few times. To tie a cord composed of nine different strands around a plumb tree, and walk off without looking back, and not divulge it, is also regarded by many as a certain cure for chronic chills.—Such superstitious delusions relative to the remedial power attributed to spells and charms have existed from time immemorial, and still exist to a very large extent even in the "Empire State of the South." It is said in Burton's life of Hume, that the poor visited the tomb of the Abbe Paris (Cemetery of St. Medard,) to bless his memory, and pray for the state of his soul, and while doing so the sick were cured, and the blind restored to sight. Admit this to be true, and admit also that many have gotten well after applying the plumb tree and mole charm, does it follow that such charms did or can have any influence in the animal economy. Not at all. The test of what is often called experience is not alone sufficient to prove the value of a remedy in the cure of disease. Because a person has been taking calomel, or some of the infinitesimal doses of Homœopathy for the cure of some disease, we must not be satisfied to say he has been cured by these agents merely, because he has got well during their administration, unless we can clearly show that their agency on the functions of the body is always or generally such as to render such a result probable; but attribute the cure to that *vis medicatrix naturæ*, which in hundreds of cases is all sufficient to resolve the disturbances of health. The number of facts in support of our last position might be augmented *ad infinitum*, but our time will not permit us to indulge further on this point. Allow me to remark, however, that this great obstacle will not be easy overcome; indeed, it can never be effectually eradicated, especially so long as physicians are kept the sole depositories of medical learning from a false idea that it would be an innovation upon the conventional rules of professional etiquette to disseminate general medical knowledge among all classes. The time has been when the profession, almost to a unit, was opposed to this plan of general diffusion of hygienic and general medical knowledge, but many of late have been made to see their error in this respect. Indeed, a new spirit is awakened throughout the profession, and if we be allowed to reason from the signs, the day is not far distant when the learned and scientific portion of the profession at least, will unite in the recommendation of this plan. The cry will be from east to west, from north to south: Let the public be enlightened; let them understand the outlines, at least, of their own system; let them have a knowledge of the laws of health; let every literary college establish a professorship of hygiene; let it be taught in every school; let every physician feel that it is his duty to instruct his friends upon the general outlines of the science of medicine and hygiene, so that they may know that disease is a morbid state, or the effects of a violation of some law of the animal economy, aud that it is cured not by tricks and charms, or by giving large or small doses either of any known or secret remedies indiscriminately, but by removing the cause, either by leaving it to the repelling or curative power of nature, or by using remedies directed by a good judgment, armed with correct views of the pathology of the disease at the time, and a thorough knowledge of the therapeutical effect of the remedies. Let this be done, and then, and not until then, will this great obstacle (the utter ignorance of the public of their own mechanism, and the law by which it is controlled, and life and health preserved,) will be removed, and along with it the thousand and one evils which are its legitimate offspring, and which have ever retarded the wheels of medical science, and have been insurmountable barriers in the way of mauy of its true votaries to usefulness and fame.

Gentlemen, this is an important and inviting theme; it involves much, and I would like to enlarge at this point, but time admonishes me that I must draw this discourse to a close. There are many things pertaining to your duties, for the want of time, I have not alluded to; many which you will find laid down in the code of medical ethics, which, if you will read carefully before you enter upon your duties, and live up to its requirements, will guide you safely over many rugged paths in the profession of your choice. "The first impressions are the most lasting." There are many, very many, good and true brethren in the profession, who will ever regret that it was not their good fortune to have read it before they took steps which they can now never retrace.

My task, gentlemen, is well nigh accomplished. It remains but to pronounce the parting words, and we separate, perhaps, to meet no more, till we meet at the great judgment bar, where we will all have to give an account for the manner in which we have improved our time and talents. I have endeavored to tell you the nature of your profession, your duties to yourself, to your patients and the public, and also to your professional brethren, as educated enlightened Christian gentlemen. Go then, gentlemen, into the untried world; a rough field of battle awaits you; put on the armor, then, that is best adapted for the conflict; determine to excel in all that is magnanimous and great; keep constantly in view the great responsibilities that rest upon you, as guardians of the public health; be true to the dignity of your heaven-born profession; be true to the reputation and prosperity of your Alma Mater; be true to yourselves, your country and your God; "get wisdom, get understanding; wisdom is the principal thing."

Let me entreat you, young gentlemen, to study the Scriptures; embrace the Christian religion, which is the "basis of every excellence—the subsistence of every virtue. It is your ornament and your strength—seek it ye who have it not—hold it fast ye who have it." It is the tree of Heaven—the gift of God to sweeten life's bitter waters ; carry it with you to what ever part of God's creation you may wander ; "trust it in prosperity ; resort to it in trouble ; shield yourselves with it in danger, and rest your fainting head upon it in death." Do this, young gentlemen, and though the world may at first look coldly upon you, and your pathway be dark, God will be with you to lead you in right paths, and you will go through life, with usefulness and honor, and to the grave with the last and blessed hope of a dying man.

I shall detain you no longer, my young brethren. Your moral and professional duties—your difficulties and their antidote are all before you. Yes, the world is before you. Your profession, with all its noble incentives to labor ; with its broad field, decked with the rich and pearly gems of science, are all before you. The hill of science, the summit of fame, and the hopes of Earth and joys of Heaven, are alike before you. Then summon to your aid all your courage ; call out your resources of energy and perseverance ; arouse the latent fires of ambition, which slumber in your breast ; buckle on your armor and prepare for the mighty conflict ; go forth with "stout arms and brave hearts," determined to make a mark on your day and generation, which no time can efface—determined to climb the "hill of science"—to mount the summit of usefulness and fame. Go forth, gentlemen, armed with such noble purposes and high resolves, and, under the blessing of Almighty God, you may live to reap a golden and glorious harvest ; you may see the wreath of fame upon your brow, and behold your name written among the few, "who were never born to die,"

> "Like those who sink to rest,
> With all their country's wishes blest."

And as Earth's crown of laurels fade from your brow, it may be replaced by a "crown of righteousness," wreathed in Heaven's richest jewels, and shineth with a lustre that fadeth not away, within the golden streets and pearly gates of the "New Jerusalem."

